# Irreversible Deactivation Pathways in Ni(II)-Catalyzed Nonalternating Ethylene–Carbon Monoxide Copolymerization

**DOI:** 10.1021/jacs.4c16468

**Published:** 2025-02-19

**Authors:** Lukas Odenwald, Lukas Wursthorn, Stefan Mecking

**Affiliations:** † Chair of Chemical Materials Science, Department of Chemistry, University of Konstanz, 78464 Konstanz, Germany

## Abstract

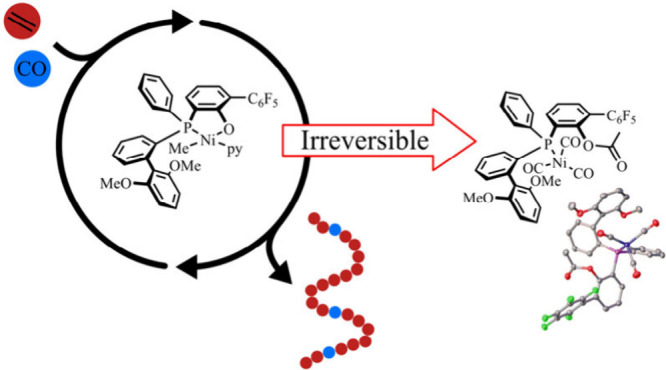

Endowing polyethylenes with photodegradability via incorporation of low densities of in-chain keto units could reduce the problematic environmental persistency of littered polymer waste. A breakthrough enabling such materials is the recent finding of nickel catalyzed nonalternating copolymerization of ethylene–carbon monoxide. We reveal irreversible catalyst deactivation pathways operative in this reaction. Reductive elimination of the common phosphinephenolate Ni(II) motif occurs with the acyl intermediates formed upon incorporation of carbon monoxide into the growing chain, as observed by low temperature NMR spectroscopy and single crystal X-ray crystallography of the isolated product. Further, we show that such decomposition pathways are generally relevant during ethylene–carbon monoxide copolymerizations under pressure reactor conditions. These findings guide the development of more stable and productive polymerization catalysts to enable the production of environmentally benign polyethylenes.

Polyethylenes are employed on an enormous scale due to their excellent material properties and low cost. As a downside, mismanaged polyethylene waste accumulates in all environments.^1,2^ Toward a more sustainable polymer economy,^3−7^ an introduction of small densities of in-chain functional groups that enable an eventual chain breakdown could reduce the problematic environmental persistency of polyethylene. Keto groups, generated by the incorporation of small amounts of carbon monoxide during chain growth, can endow the material with desirable photodegradability.^8^ The required nonalternating copolymerization^9−11^ had been long sought for, and was recently achieved utilizing advanced phosphinephenolate Ni(II) complexes.^12−16^ Copolymerization of ethylene at only 5 atm overall pressure with low concentrations of carbon monoxide yields polyethylenes with isolated keto groups. Due among others to their high molecular weights (up to *M*_w_ 400,000 g mol^–1^; *M*_n_ 200,000 g mol^–1^) these polymers are processable and on par in their mechanical properties with commercial high-density polyethylene (HDPE).^16^ At the same time, they are photodegradable.

In olefin–CO copolymerizations, chain growth is slowed by chelating coordination of keto repeat units of the growing chain. This reversible deactivation mode was established comprehensively for the case of alternating copolymerizations with cationic Pd(II) catalysts by Brookhart et al.^17,18^ For the case of neutral phosphinephenolate Ni(II) catalysts, theoretical studies show that the opening of such chelates is also a decisive step. Overall, for reversible deactivation of catalytic insertion polymerization by polar vinyl monomers and carbon monoxide, respectively, a fairly comprehensive qualitative and in some cases even quantitative understanding exists.^19−22^ By contrast, although irreversible catalyst deactivation pathways are equally important and perhaps ultimately more relevant for catalyst performance, they have rarely been elucidated.^23,24^

We now report on the identification of a carbon monoxide-specific deactivation pathway of relevant ethylene polymerization catalysts, observed in stoichiometric and preparative pressure reactor experiments. This understanding can aid in the rational choice of polymerization conditions and the development of more stable catalysts to enable the production of environmentally benign polymers.

Complex **1** as a catalyst precursor enables the synthesis of high molecular weight keto-modified polyethylene materials.^16^ Motivated by these catalytic properties, we explored the fundamental reactivity of **1** toward CO.
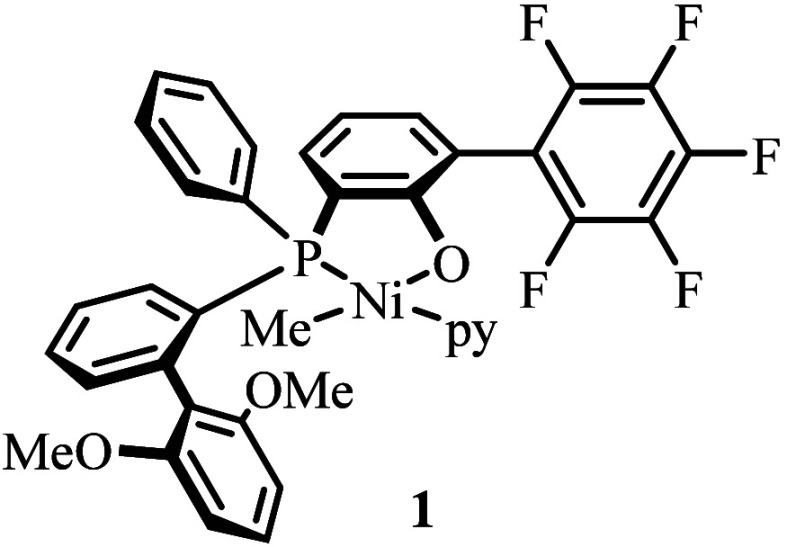


Exposure of **1** to ^13^CO in methylene chloride-*d*_2_ solution at low temperatures (−78 to −30 °C) resulted in the formation of new species **2** and **3** as observed by NMR spectroscopic monitoring (Figure 1 and Figures S2 and S3 in the Supporting Information (SI)). Compound **2** features characteristic ^1^H resonances of 1.68, 3.46, and 4.20 ppm as well as ^13^C resonances of 264.3 and 35.4 ppm. For **3**, ^1^H characteristic resonances arise at 3.67, 2.60, and 1.45 ppm as well as ^13^C resonances at 195.6, 167.8, and 19.6 ppm. With excess CO, a virtually complete conversion of **1** is achieved (SI, Figure S2). Upon keeping a sample overnight at −30 °C, an increase in the amount of **3** at the expense of **2** is observed (Figure S4). A remarkably low field ^13^C resonance at 264.3 ppm identifies **2** as the acyl complex expected to form by the insertion of carbon monoxide into the Ni–Me bond of **1**. Such a low field chemical shift has been observed for other acyl complexes and was ascribed to a carbenoidal bonding character^25−27^ (cf. Figure S5). The NMR signature of the organic carbonyl moiety in compound **3** was identified as a phenolate acyl ester. The ^31^P shift suggests that the phosphine is still coordinated to the metal. Upon exposure to excess CO for multiple days, the phosphine in **3** is displaced, yielding free, uncoordinated phosphine ester **4** (identified by comparison of ^1^H and ^31^P NMR signals to those of a genuine sample, cf. Figure S12).

**Figure 1:**
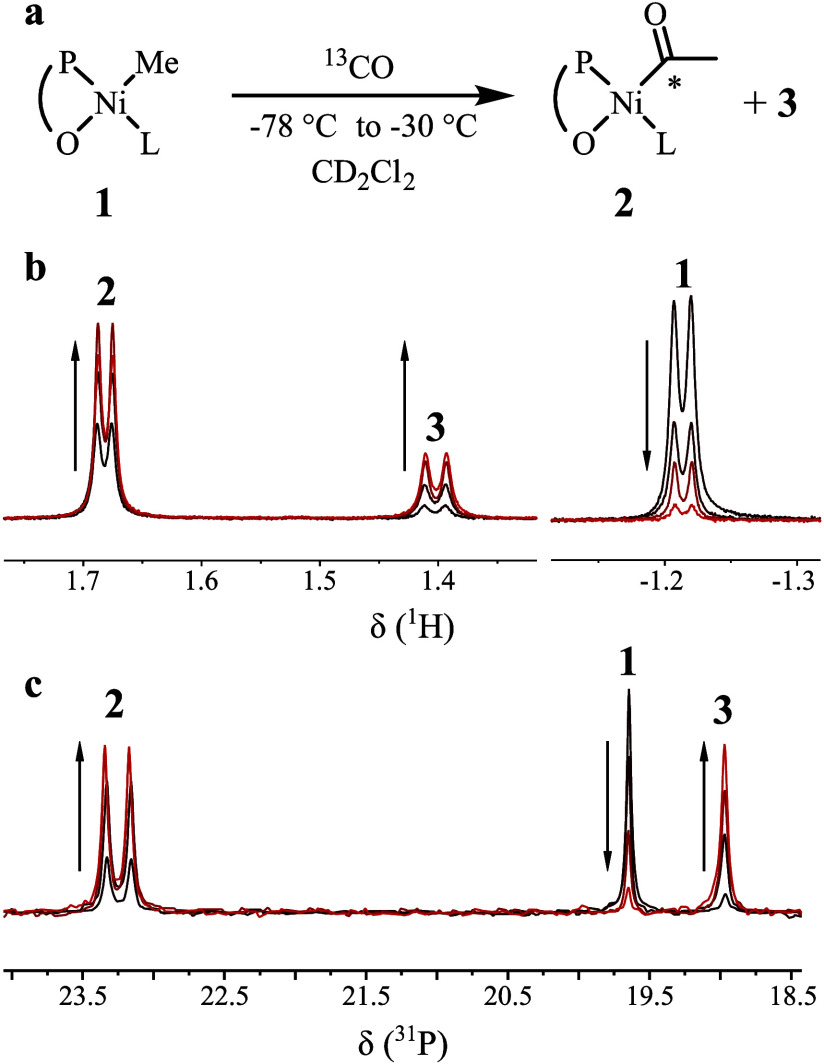
NMR spectroscopic observation of the reaction of **1** with increasing amounts of carbon monoxide added portion-wise; final pressure: 1.5 atm ^13^CO (see SI for details of procedure). Arrows indicate the evolution of the signals with increasing amounts of carbon monoxide. (a) Reaction scheme and formed species. (b) ^1^H NMR spectra (CD_2_Cl_2_, −30 °C) showing the region of α-carbonyl methyl and nickel methyl groups. (c) ^31^P NMR spectra (CD_2_Cl_2_, −30 °C).

Isolation from the reaction solution by crystallization and analysis by single crystal X-ray diffraction (Figure 2b) unambiguously identified **3** as the phenol acetate, coordinated to Ni via the phosphine moiety. In line with a nickel(0) oxidation state, **3** possesses a tetragonal coordination geometry. This further confirms that **3** is the product of reductive ligand elimination from the acyl intermediate (Figure 2a).

**Figure 2:**
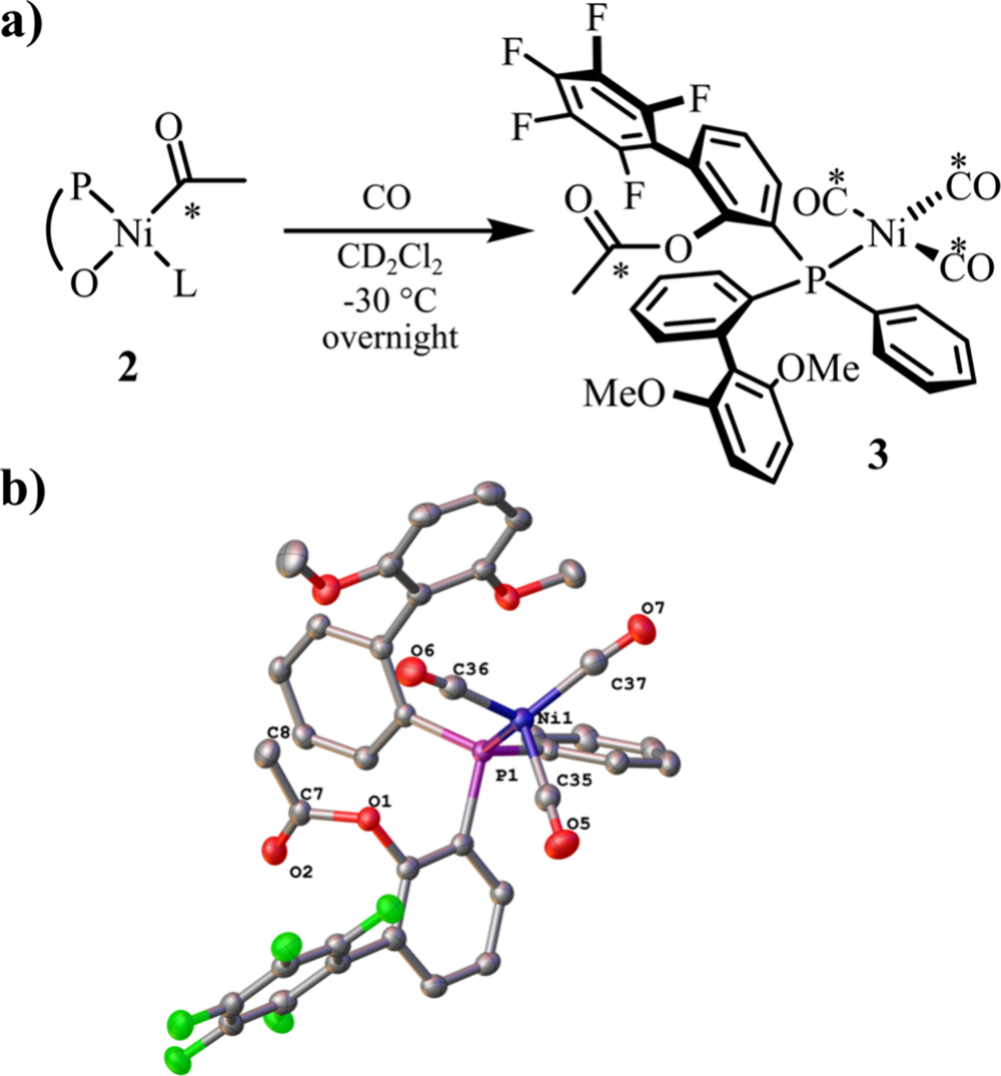
(a) Formation of **3** by reductive elimination from **2**. (b) Solid state structure of **3** as obtained by single crystal X-ray diffraction. Thermal ellipsoids drawn at 50% probability level. Hydrogen atoms are omitted for clarity.

The carbenoidal character of the acyl species, as identified by the low-field chemical shift of its ^13^C resonance, makes the carbonyl more accessible for reactions with nucleophiles. This can promote an internal attack of the phenolate facilitating reductive elimination of the acyl ligand.

The reductive elimination reaction is strongly suppressed by excess Lewis base. Exposure of **1** to 2 bar of ^13^CO in the presence of 20 equiv of pyridine at −30 °C resulted in 60% conversion to the acyl species **2** without detectable amounts of **3**, contrasting the behavior without additional pyridine (vide supra and see SI Figure S6). Hence, we propose an acyl carbonyl complex as starting point of the reductive elimination pathway, in line with the suggestion of a five-coordinated intermediate for related systems brought forward previously.^26,28,29^

Eyring analysis of the reaction from **2** to **3** in CO-saturated toluene-*d*_8_ ([CO] ≈ 7.5 mM^30,31^) in the temperature range from 238 to 269 K reveals an activation enthalpy of Δ*H*^⧧^ = 12.4 (0.7) kcal mol^–1^ and an activation entropy of Δ*S*^⧧^ = −24.3 (2.7) cal K^–1^ mol^–1^ (see Figures S7 and S8 in the SI). The negative activation entropy indicates an ordered intermediate and in this sense agrees with the assumed occurrence of a 5-membered intermediate formed upon coordination of CO to complex **2** (*vide supra*). Extrapolation to polymerization temperatures of 90 °C gives an activation energy of Δ*G*^⧧^ = 21.3(1.7) kcal mol^–1^, which agrees qualitatively with the observation that this deactivation route is accessible under pressure reactor conditions (*vide infra*).

These findings raise the question of whether intermediates of catalytic chain growth can also undergo such an elimination and whether this is a relevant deactivation pathway operative during polymerization. To this end, **1** was exposed to ethylene (8.5 atm) and ^13^C labeled carbon monoxide (0.5 atm) in a pressure reactor at a typical polymerization temperature of 90 °C. Analysis of the solution separated from the formed solid (see Figure S16 for characterization of the formed solid) by NMR methods showed a distinct ^31^P NMR resonance at −22 ppm. A comprehensive comparison of ^1^H, ^13^C, and ^31^P NMR data to the spectroscopic data of a genuine sample of **4** prepared separately for this purpose (see SI for details of synthesis and characterization) reveals the minor portion of the ^31^P signal at −22.53 ppm arises from this phosphinephenol-acetate, formed from the catalyst precursor (cf. Figure 3b). The major peak of this ^31^P NMR, at −22.30 ppm, corresponds to a compound containing at least two carbonyl groups as concluded from the doublet structure of the corresponding ester ^13^C signal at 170.0 ppm. This doublet was attributed to a ^13^C–^13^C coupling of the neighboring carbonyls (resulting from ^13^C labeling). Comparison to an independently prepared genuine sample of **5** (see the SI for details of synthesis and characterization) reveals this to be a phenol ester formed by reductive elimination of the growing polymer chain to the phosphinephenolate ligand of the catalyst. Note that phosphine oxides as oxidation products of the ligand can also be observed (see Figure S15).

**Figure 3:**
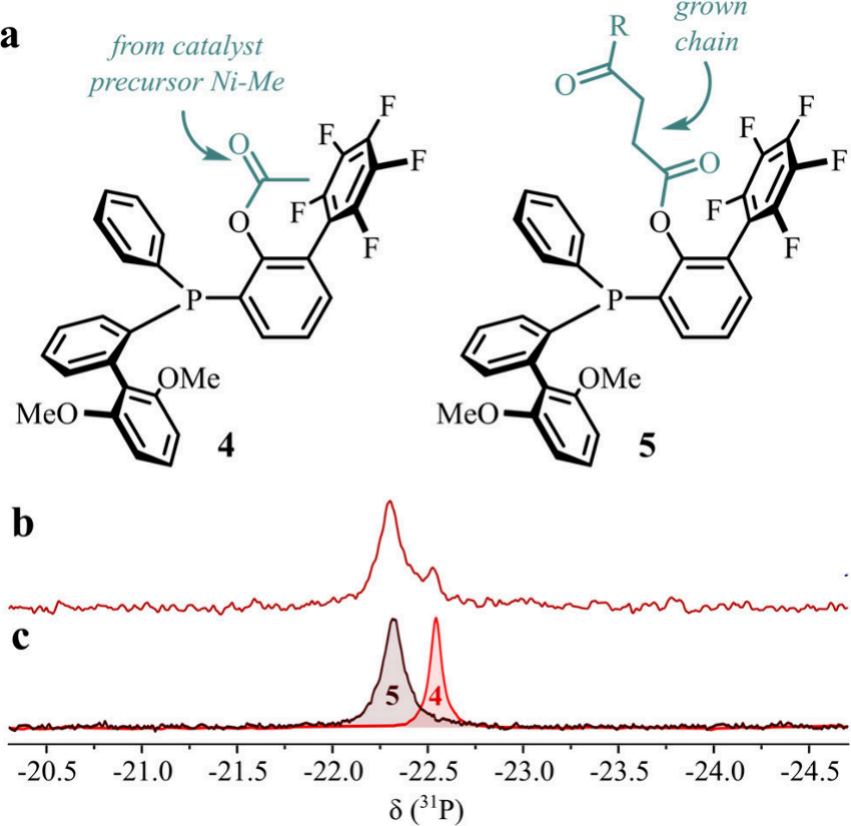
Key ^31^P NMR resonances, observed in reaction solutions from pressure reactor experiments, and comparison to genuine samples of **4** and **5**. (a) Structure of **4** and **5**. (b) ^31^P NMR spectrum of the solution from pressure reactor experiments. (c) Superimposed ^31^P NMR spectra of genuine samples of **4** and **5**, respectively.

Carbon monoxide is generally a challenging substrate, as it is a strongly binding ligand and also a reducing agent for transition metals. Thus, it is all the more relevant to understand which pathways affect the catalyst performance during polymerization. Our findings identify reductive elimination of acyl intermediates to a chelating phosphinephenolate ligand as an irreversible decomposition pathway. The particular bonding character^22^ of acyls which combine a Ni–C bond with the electrophilicity of the carbonyl motif can promote nucleophilic internal attack.^23^ Experimental observations under pressure reactor conditions and thermodynamic data underline this pathway as accessible under polymerization conditions.

The actual extent to which catalyst performance is limited by the pathway identified here is expected to depend on the CO concentration. Notably in terms of feasibility of nonalternating copolymerization, the catalyst formed from **1** as catalyst precursor features a lifetime of several hours in pressure reactor polymerizations to keto-polyethylenes with desirable low CO incorporations of ca. 1 mol %.^16^

The mechanistic insights obtained provide possible guidelines to improve catalyst longevity via catalyst design and the polymerization reaction conditions. A rapid trapping of acyl intermediates, as they form upon CO insertion, by ethylene^20^ followed by a rapid olefin insertion (*k*_ins_^E-acyl^, pathway a in Figure 4) along with a higher activation barrier of the reductive elimination (pathway b in Figure 4) could disfavor the type of irreversible deactivation revealed here. Thus, reaction conditions may determine not only the composition and microstructure of the polymer product but also the catalyst lifetime. Screening protocols in the evaluation of novel catalyst structures can be amended by monitoring of the acyl reductive elimination products upon CO exposure. A further understanding of these mechanisms is clearly a desirable and worthwhile objective toward the aim of enabling more environmentally benign polyolefin materials.

**Figure 4:**
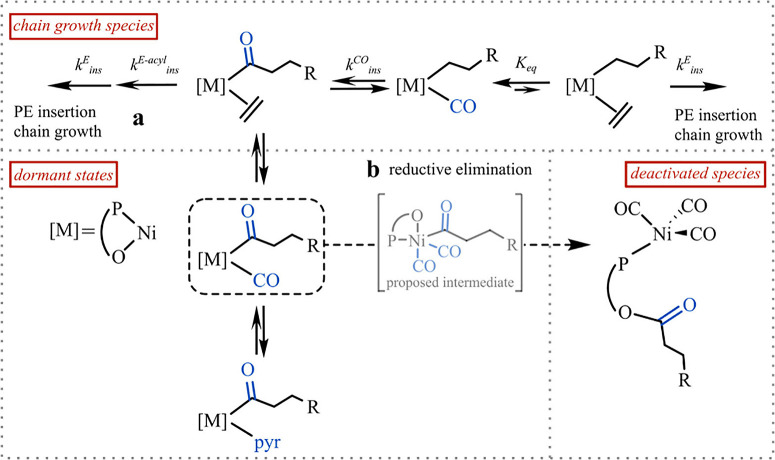
Possible impact of competitive monomer coordination on suppression of the deactivation route was identified.

We anticipate that our insights will be instructive for the design of novel catalysts for in-chain functionalized polyethylene materials.

## Supporting Information Available

Experimental procedures, characterization data, spectra (PDF)
