# Supplementary material for: Irreversible Deactivation Pathways in Ni(II)-Catalyzed Nonalternating Ethylene–Carbon Monoxide Copolymerization
Source: J Am Chem Soc. 2025 Feb 19;147(9):7182–6. doi: 10.1021/jacs.4c16468 (PMC11887445; doi:10.1021/jacs.4c16468)
Supplement: Supplementary file 1 — ja4c16468_si_001.pdf [file ja4c16468_si_001.pdf]

Supporting Information

Irreversible Deactivation Pathways in Ni(II)-Catalyzed Non-alternating Ethylene-Carbon Monoxide Copolymerization

Lukas Odenwald, Lukas Wursthorn and Stefan Mecking

Chair of Chemical Materials Science, Department of Chemistry, University of Konstanz, 78457 Konstanz, Germany

Unless stated otherwise all polymerizations and manipulations were performed under nitrogen atmosphere using standard Schlenk and glove box techniques.

NMR spectra were recorded on a Bruker Avance III 400 spectrometer, a Bruker Avance III HD 400, or a Bruker Avance III 600 spectrometer. ^{1}H, ^{13}C chemical shifts were referenced to the solvent signals, ^{31}P and ^{19}F were referenced by absolute referencing. NMR spectra are reported as follows: chemical shift (δ in ppm), multiplicity, coupling constant (Hz), and integration. Multiplicities were assigned as follows or combinations thereof: s: singlet, d: doublet, t: triplet, m: multiplet, v: virtual, br: broad. In addition to 1D-NMR experiments, 2D-NMR experiments (^{1}H-^{1}H COSY, ^{1}H-^{13}C HSQC, ^{1}H-^{13}C HMBC) were carried out for full assignment. NMR data was post-processed using MestReNova software.

For X-ray diffraction analysis a suitable crystal was selected and fixated on a glas fiber with grease and mounted on on a STOE IPDS 2T diffractometer. The crystal was kept at 100 K during data collection. Using Olex2 [1], the structure was solved with the SHELXT[2] structure solution program using Intrinsic Phasing and refined with the SHELXL[3] refinement package using Least Squares minimisation. Graphical representation was created by the ORTEP-3 V2.02. for the Windows XP software package.[4]

Solvents were dried and degassed using standard laboratory techniques. Ethylene (3.5 grade) and carbon monoxide (4.7 grade) were supplied by Air Liquide and used as received. ^{13}CO with an isotopic purity of >99 % was purchased from Eurisotop. All deuterated solvents were supplied by Eurisotop. If not stated otherwise, solvents were used in technical grades as received. 2-(2-[2,6-$(MeO)_2C_6H_3$]C_6H_4)(Ph)P-6-C_6F_5-C_6H_4OH (Phosphinophenole **1'**) and its respective precatalyst **1**[5] as well as levulinic anhydride[6] were synthesized according to reported procedures. Dimethylaminopyridine was purchased from Sigma-Aldrich Chemie GmbH; pyridine and acetic anhydride from Acros Organics, and used as received.

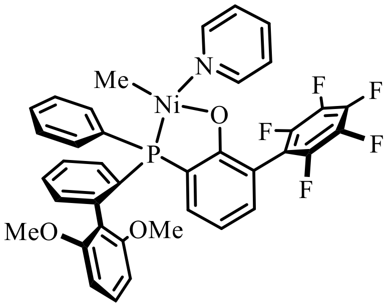

1

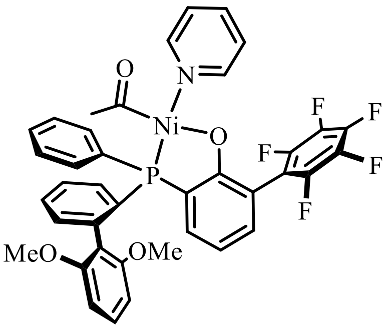

2

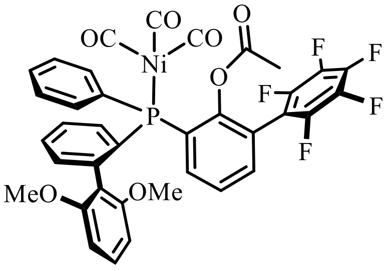

3

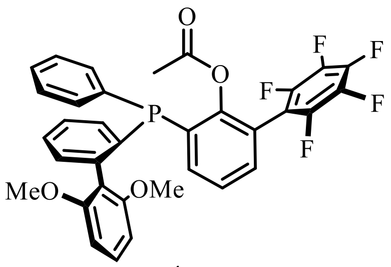

4

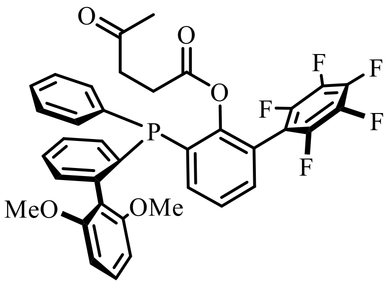

5

Figure S1. Overview of the compounds discussed and their structure.

1. Mechanistic NMR spectroscopic studies

Procedure: 1 (10 µmol, 7.3 mg) was dissolved in 500 µL of deuterated methylene chloride in a high-pressure NMR tube. Initial spectra were recorded at -30 °C. The NMR tube was capped with tubing which allowed evacuation and flushing with nitrogen and the addition of carbon monoxide with syringes. The NMR tube was cooled to -78 °C and evacuated to remove the nitrogen atmosphere. With a syringe, 8 mL of ^{13}C labeled carbon monoxide were added (note: the headspace of the tubing is also filled, and not the complete 8 mL of carbon monoxide are added to the NMR tube). The NMR tube was inserted into a precooled NMR spectrometer (-30 °C) and spectra were recorded. To increase the amount of carbon monoxide, the tube was once again removed from the spectrometer and directly cooled to -78 °C. The tubing was connected, the headspace was evacuated, and another 8 mL of carbon monoxide were added via syringe. This process was repeated multiple times. Finally, to achieve full conversion, the NMR tube was pressurized with 1.5 bar of ^{13}CO. The reaction progress was then monitored overnight

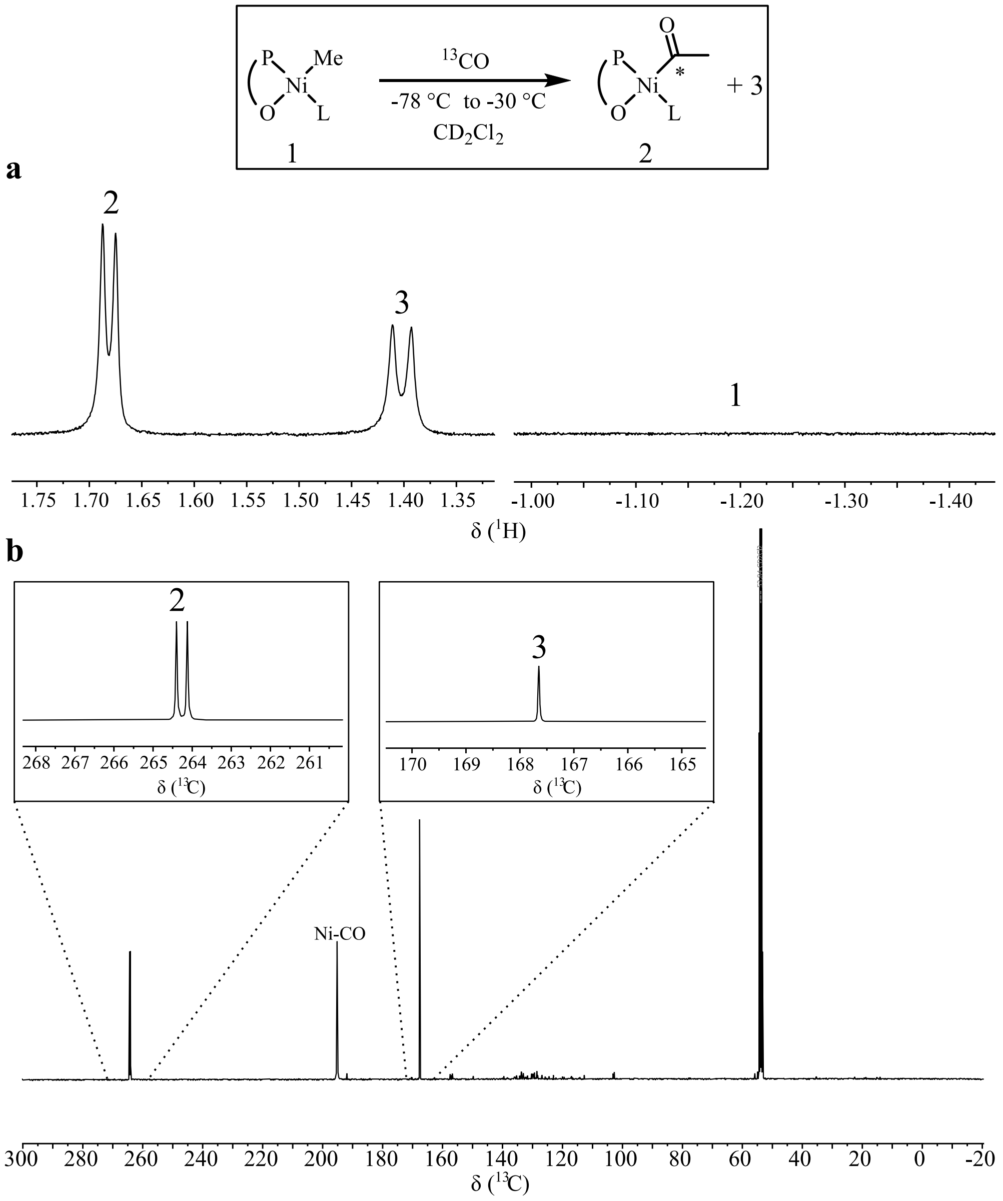

Figure S2. Complex **1** treated with 1.5 bar ^{13}C labeled carbon monoxide. **a:** Details of the ^{1}H NMR (CD_2Cl_2, -30 °C) spectrum illustrating full conversion of **1** to a mixture of species **2** and **3** . **b:** ^{13}C NMR spectrum (CD_2Cl_2, -30 °C) of the mixture. Expansions show carbonyl signals of species **2** and **3.**

Compound **2**:

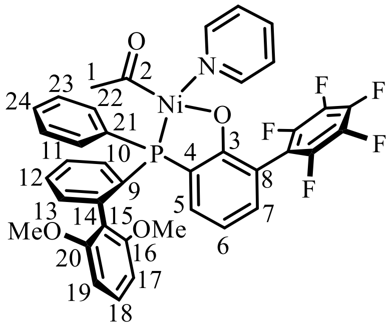

^{1}H NMR (400 MHz, CD_2Cl_2, 243 K) δ[ppm] = 8.63 (s, 2H, o-Py), 8.14 – 8.05 (m, 2H, H-22), 7.79 (s, 1H, p-Py), 7.53 – 7.13 (m, 8H, m-Py, H-10, -11, -12, -13, -23, -24), 7.08 – 6.97 (m, 2H, H-18, -5), 6.83 (d, 1H, *J* = 7.3 Hz, H-7), 6.63 (d, 1H, *J* = 8.5 Hz, H-19 or H-17), 6.23 – 6.14 (m, 2H, H-19 or H-17, H-6), 4.20 (s, 3H, -OMe), 3.46 (s, 3H, -OMe), 1.68 (d, 3H, *J* = 5.0 Hz, H-1).

^{13}C NMR (151 MHz, CD_2Cl_2, 243 K) δ[ppm] = 264.26 (d, *J* = 27.7 Hz, C-2), 170.26 (d, *J* = 22.1 Hz, C-3), 157.49 (C-20 or C-16), 156.74 (C-16 or C-20), 149.78 (o-Py), 139.60 (d, *J* = 9.8 Hz, C-14), 138.89 – 137.79 (m, p-Py), 134.04 – 133.42 (m, C-7, -22), 133.30 – 132.99 (m, C-5), 132.25 (d, *J* = 34.8 Hz, C-9), 129.83 – 129.55 (m, C-24) 129.40 (C-18), 128.51 (d, *J* = 10.4 Hz, C-23), 124.48 (m-Py), 119.71 (d, *J* = 52.2 Hz, C-4), 116.95 (d, *J* = 3.2 Hz, C-15), 114.36 – 113.99 (m, C-8), 112.73 (d, *J* = 7.9 Hz, C-6), 103.02 (C-19 or C-17), 102.74 (C-17 or C-19), 55.83 (-OMe), 54.90 (-OMe), 35.36 (dd, *J* = 18.1, 5.4 Hz, C-1). Signals of C-21, -10, -11, -12, -13 and the perfluorophenyl group were not resolved.

^{31}P NMR (162 MHz, CD_2Cl_2, 243 K) δ[ppm] = 23.21 (d, *J* = 27.6 Hz)

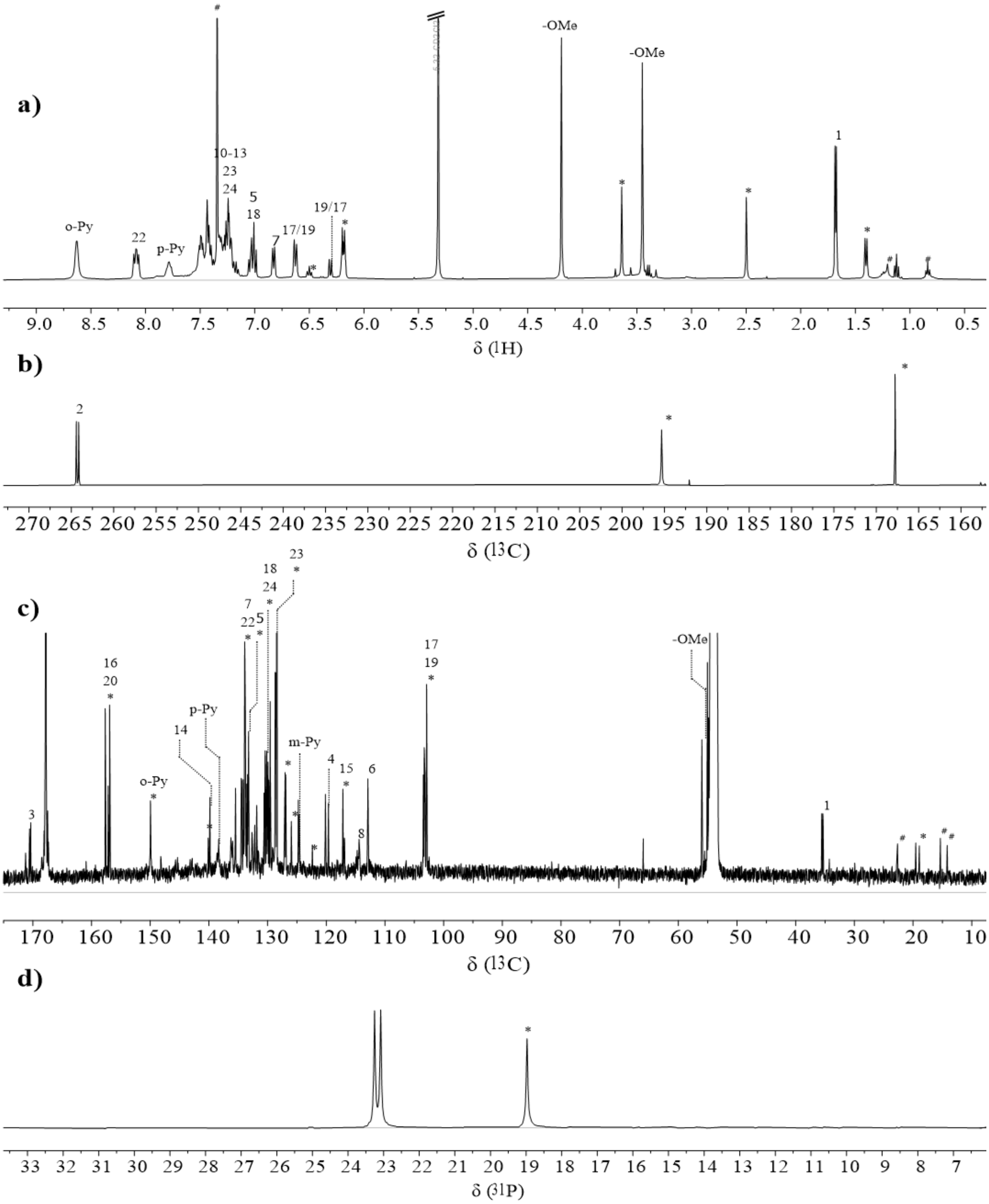

Figure S3. NMR data (25 °C, CD_2Cl_2) of compound **2**. **a:** ^{1}H NMR spectrum. **b:** ^{13}C NMR spectrum low field region. **c:** ^{13}C NMR spectrum high field region. **d)** ^{31}P NMR spectrum. *Resonances of compound **3** (cf. Figure S9)

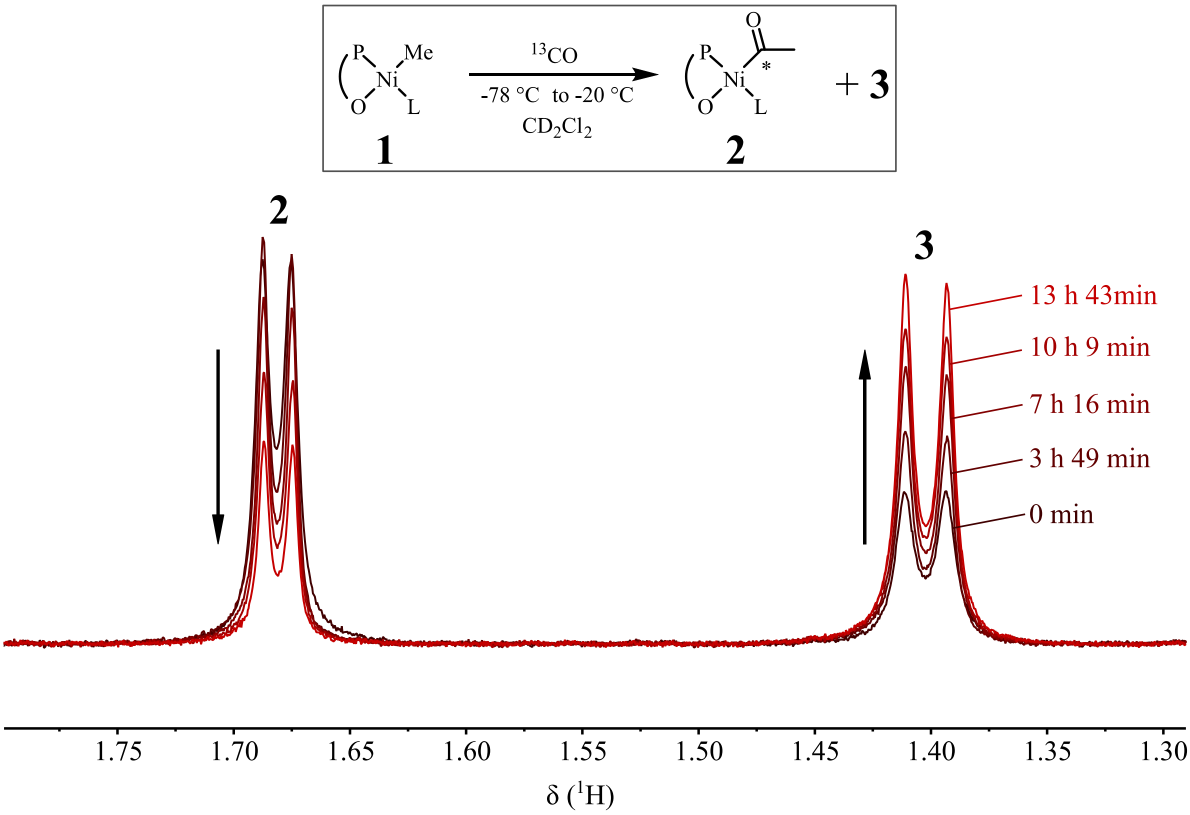

Figure S4. Evolution of ^{1}H NMR spectra of complexes **2** and **3** over time. Shown are the methyl groups adjacent to the corresponding carbonyl moiety (acyl complex or phenol ester) of compounds **2** and **3**. Arrows indicate the evolution of the signals over time. Depicted times refer to the interval after the last addition (1.5 bar) of carbon monoxide (cf. procedure).

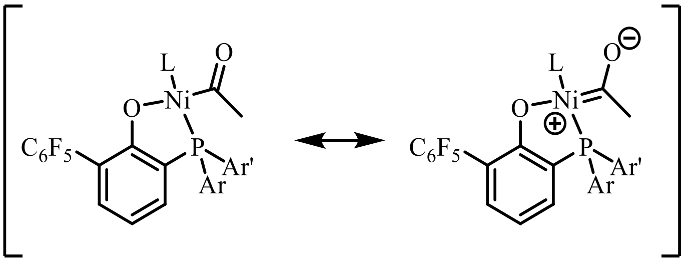

Figure S5. Resonance structures of an acyl complex with a carbinoidal character.

2. Mechanistic NMR spectroscopic studies in the presence of additional pyridine

Procedure: **1** (10 µmol, 7.3 mg) was dissolved in a mixture of 500 µL of deuterated methylene chloride and 16 µL of pyridine (20 eq) in a high-pressure NMR tube. Initial spectra were recorded at -30 °C. The NMR tube was capped with tubing which allowed evacuation and flushing with nitrogen and the addition of carbon monoxide. The NMR tube was cooled to -78 °C and evacuated to remove the nitrogen atmosphere. The tube was pressurized with 2 bar of ^{13}C-labelled carbon monoxide. The NMR tube was inserted into a precooled NMR spectrometer (-30 °C) and spectra were recorded. For comparison an identical experiment without addition of pyridine was conducted.

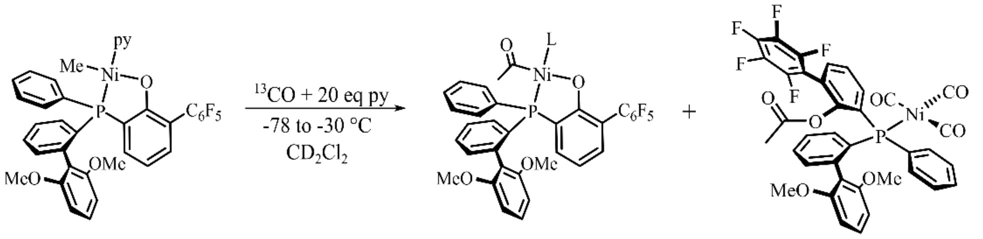

1 **2** **3**

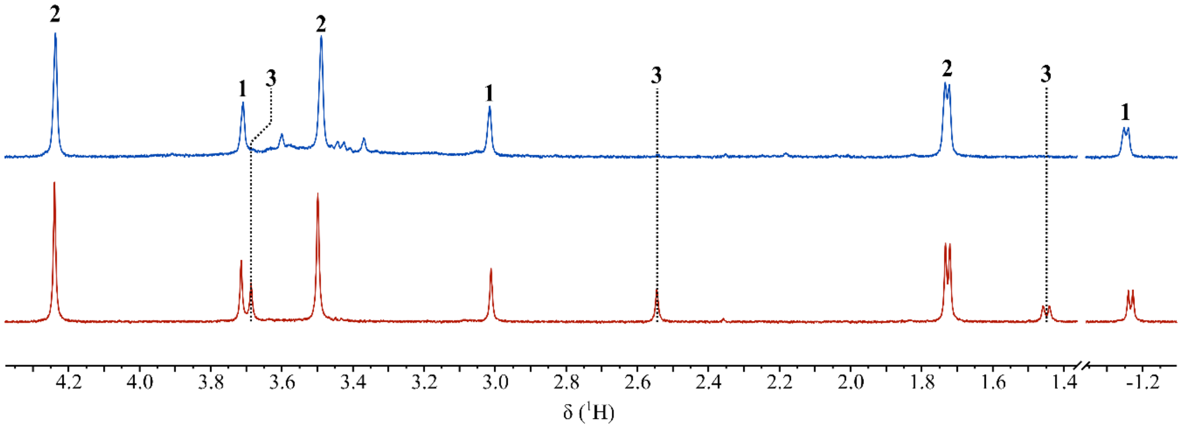

Figure S6. ^{1}H NMR spectrum (CD_2Cl_2, -30 °C) of complex **1** with (blue) and without (red) the addition of 20 equivalents of pyridine treated with 2 bar ^{13}C labeled carbon monoxide including assignment of distinct signals of species **2** and **3**. Addition of pyridine appears to inhibit the reductive elimination and the formation of **3**.

3. Eyring analysis

A solution of Ni-Me complex **1** (1 µmol) in Toluene-d_8 (0.5 mL) was placed in a J-Young NMR tube. The tube was connected to a Schlenk-line, placed under a nitrogen atmosphere and cooled to -78 °C using dry ice. To ensure full CO saturation of the solution, CO gas was slowly bubbled through the solution (5 mL min^{-1}, to avoid overflowing and warming of the solution by the room temperature gas) at -78°C for 15 min. The NMR probe was precooled to the desired reaction temperature and the precise temperature was measured before and after the reaction using a high-precision thermocouple placed inside a toluene-filled NMR tube. The sample was inserted into the spectrometer, taking care that the sample does not warm up during the transfer to prevent premature reaction. ^{1}H NMR spectra were recorded in five minute intervals until full conversion of **2** was observed. The integral of the methyl signal were used to follow and quantify the reaction progress. Reaction rates were obtained by mono-exponential fitting of the time-concentration curves. Plotting ln(k/T) vs. 1/T of reactions at different temperatures in an Eyring plot gives the activation Enthalpy as the slope and the activation entropy as the y-intercept. Uncertainties of $\Delta H^‡$ and $\Delta S^‡$ were calculated from the standard deviations of the slope and the y-intercept, respectively. Because saturation concentrations vary only slightly in the investigated temperature range (max. 5 %, 7.3 mM at 238 K vs. 7.7 mM at 269 K)[7,8], its effect was considered negligible. Linear fitting, standard deviation and confidence intervals were calculated by unweighted, least square fitting using OriginPro.

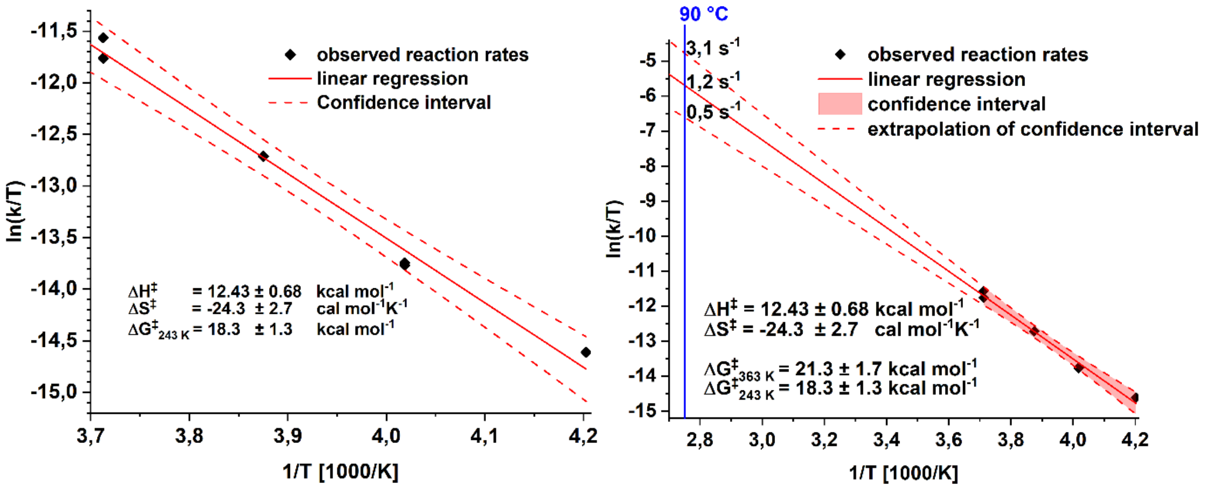

Figure S7. Eyring analysis of the reductive elimination from **2** to **3** in CO-saturated toluene-d_8 across a temperature range of 31K from 238 K to 269 K (left). Extrapolation to polymerization temperature of 90°C (right). Reactions were monitored by ^{1}H NMR spectroscopy, and rates determined by exponential fitting of the reaction profiles.

$$ln\frac{k}{T} = -\frac{\Delta H^‡}{RT} + \frac{\Delta S^‡}{R} + ln\frac{k_B}{h}$$

Calculation of the activation enthalpy from the slope:

$$\frac{d\ln\frac{k}{T}}{d\frac{1}{T}} = -\frac{\Delta H^‡}{R} \qquad \Delta H^‡ = -\frac{d\ln\frac{k}{T}}{d\frac{1}{T}} * R = 12.43 \pm 0.68\, kcal\, mol^{-1}$$

Calculation of the activation enthalpy from the y-intercept:

$$y_0 = \frac{\Delta S^‡}{R} + ln\frac{k_B}{h} \qquad \Delta S^‡ = \left(y_0 - ln\frac{k_B}{h}\right) * R = -24.3 \pm 2.7\, cal\, mol^{-1}K^{-1}$$

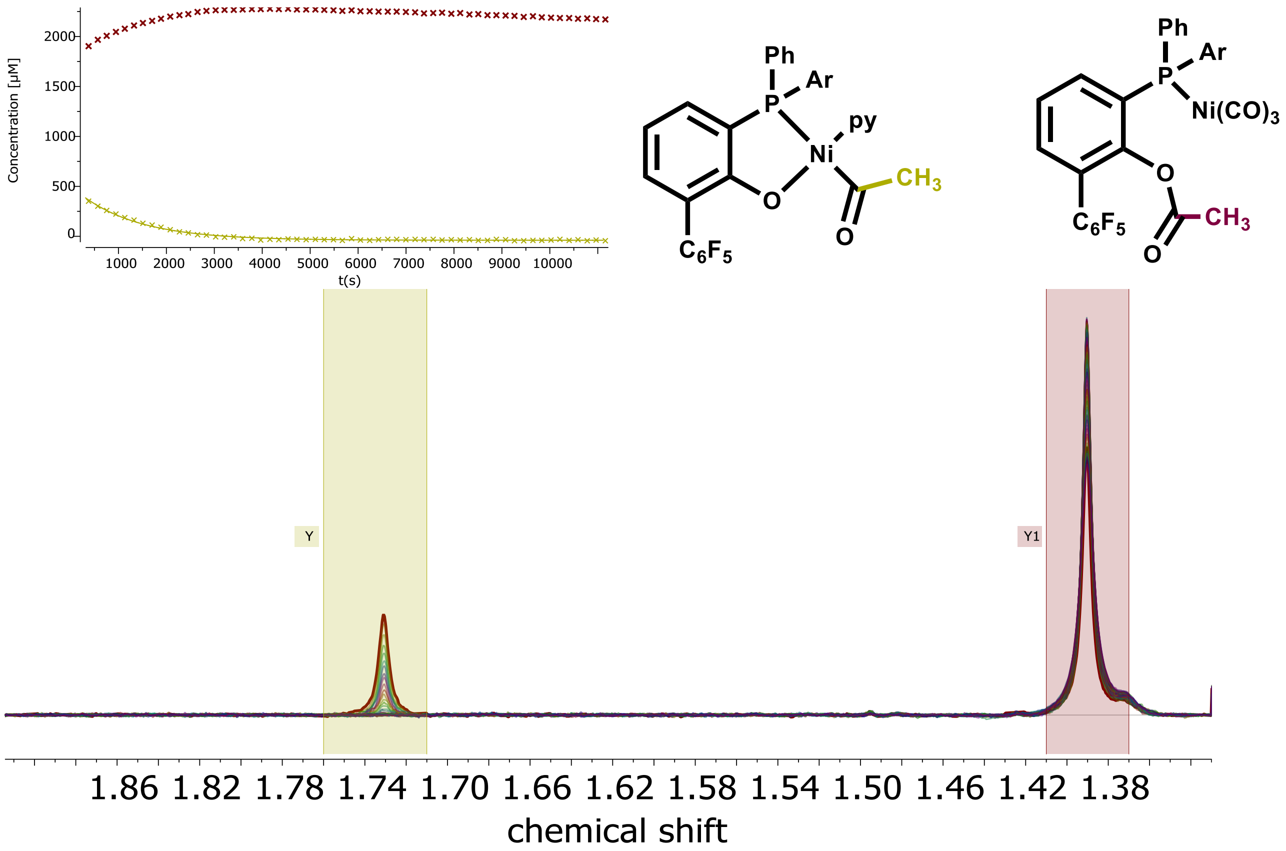

Figure S8. Exemplary determination of the reaction rate by integration of the ^{1}H-NMR acyl signal of **2** in CO-saturated toluene-d_8 at 258 K. ^{1}H-NMR spectra of the reaction mixture, collected in 5-minute intervals (bottom). Integrals of the acyl signal and mono-exponential fitting of concentration-time curves, giving the rate constant k (7.77 * 10^{-4} s^{-1}). Note that **1** had completely reacted at t_0.

4. Reference Compounds

Compound **3**:

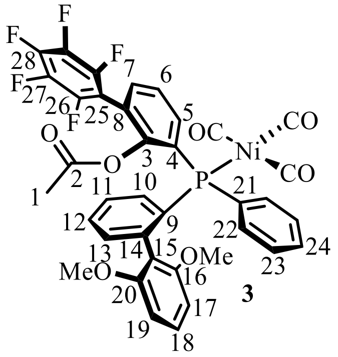

3

A solution of **1** (68.3 µmol, 50 mg) in 1.5 mL dry and degassed methylene chloride was cooled to -40 °C. Vacuum was applied to remove the nitrogen atmosphere. The flask was flushed with 1 atm of carbon monoxide. The gas supply was disconnected, and the flask was sealed and stored for 12 h at -30 °C. The solvent was removed under reduced pressure. The residue was washed with pentane (3 mL, 3x) and dried under reduced pressure to yield the product with impurities (ca 10 %) of compound **4** (vide infra).

Single crystals for X-ray crystallography were grown from a saturated methylene chloride solution via gas phase diffusion with pentane at -30 °C.

A sample for elemental analysis was recrystallized by cooling a saturated toluene/pentane solution to -30 °C.

^{1}H NMR (400 MHz, CD_2Cl_2, 243 K) δ[ppm] = 7.65 (m, 2H, H-22), 7.55 – 7.40 (m, 5H, H-10, -12, -13, -23, -24), 7.38 – 7.14 (m, 5H, H-6, -7. -11, -13, -18), 6.64 (d, 1H, *J* = 8.4 Hz, H-19 or H-17), 6.57 (td, 1H, *J* = 8.0, 1.7 Hz, H-5), 6.33 (d, 1H, *J* = 8.4 Hz, H-17 or H-19), 3.67 (s, 3H, -OMe), 2.60 (s, 3H, -OMe), 1.45 (s, 3H, H-1).

^{13}C NMR (151 MHz, CD_2Cl_2, 243 K) δ[ppm] = 195.64 (Ni-CO), 167.80 (C-2), 157.87 (C-16 or C-20), 157.55 (C-20 or C-16), 149.02 (C-3), 140.72 (d, *J* = 23.1 Hz, C-14), 136.49 (d, *J* = 30.0 Hz, C-4), 135.88 (C-12), 135.43 – 134.73 (m, C-22), 134.42 (d, *J* = 8.9 Hz, C-13), 133.42 (d, *J* = 32.4 Hz, C-21), 132.70 (d, *J* = 4.9 Hz, C-5), 132.24 (C-7), 130.98 (d, *J*=33.7 Hz, C-9), 130.34 (m, C-10/-24), 130.12 (C-18), 129.01 (d, *J* = 9.9 Hz, C-23), 127.32 (d, *J* = 4.7 Hz, C-11), 126.12 (d, *J* = 5.9 Hz, C-6), 122.88 (C-8), 117.88 (d, *J* = 5.3 Hz, C-15), 103.98 (C-17 or C-19), 103.79 (C-19 or C-17), 55.13 (-OMe), 53.66 (-OMe), 19.57 (C-1), signals of the perfluorophenyl group were not resolved.

^{31}P NMR (162 MHz, CD_2Cl_2, 243 K) δ[ppm] = 20.05.

Elem. Anal. found (calcd) for toluene adduct of **3** ($C_{37}H_{24}F_5NiO_7P \cdot C_7H_8$): C, 61.10 (61.64); H, 4.06 (3.76).

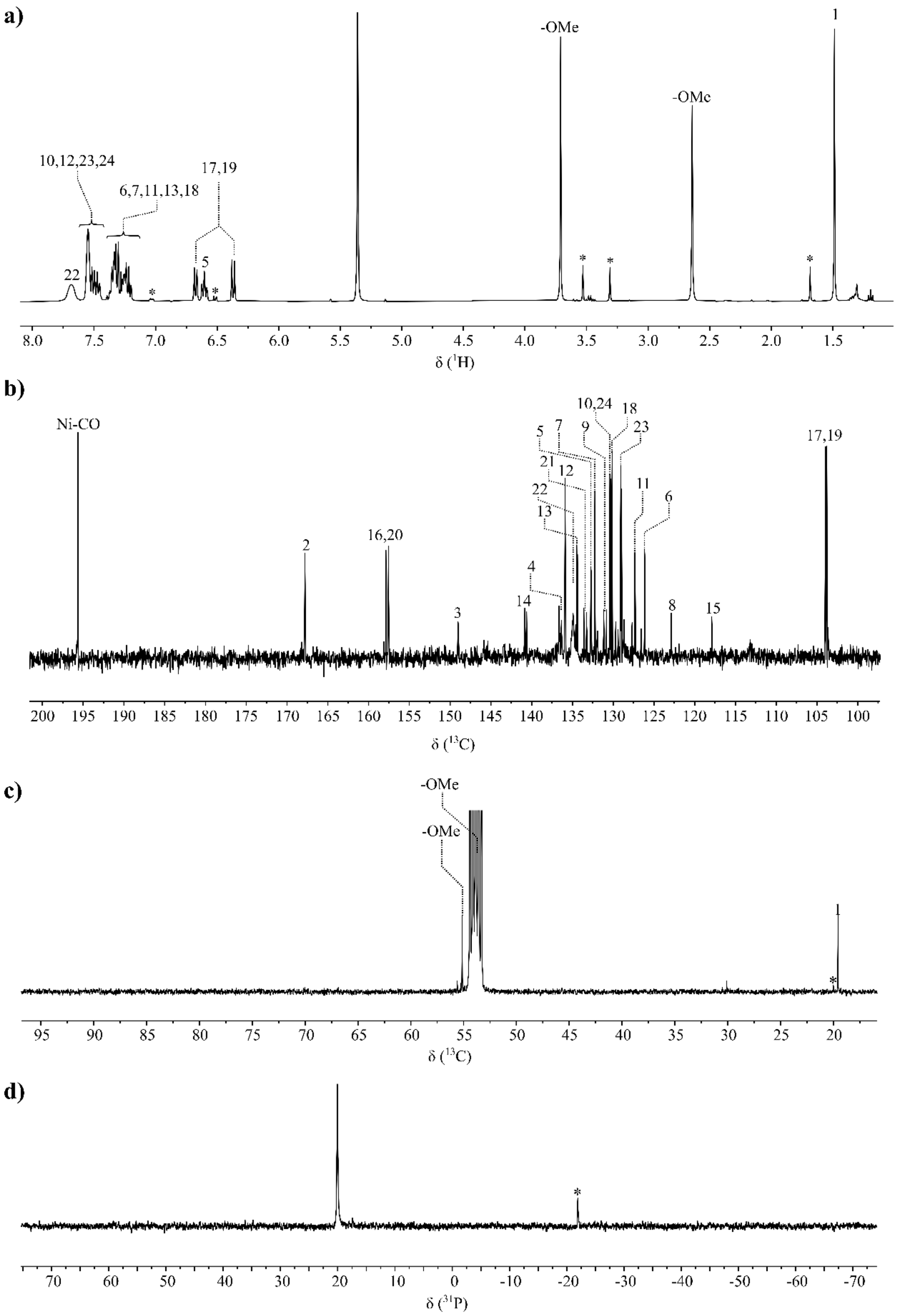

Figure S9. NMR data (25 °C, CD_2Cl_2) of compound **3**. **a:** ^{1}H NMR spectrum. **b:** ^{13}C NMR spectrum low field region. **c:** ^{13}C NMR spectrum high field region. **d)** ^{31}P NMR spectrum. *Resonances of compound **4** (cf. Figure S10)

Compound **4**:

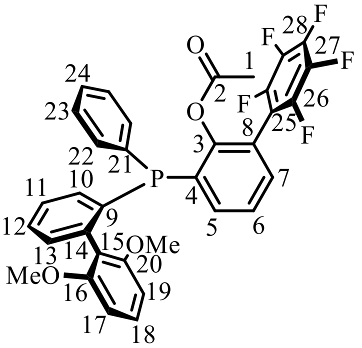

654.61 g/mol
$C_{36}H_{32}F_5O_4P$

To a solution of **1'** (1 eq, 86.2 µmol, 50 mg) and dimethylaminopyridine (10 mol%, 8.6 µmol, 1.1 mg) in 0.5 mL of dry pyridine acetic anhydride (2 eq, 172 µmol, 17.6 mg, 16.3 µL) was added at room temperature. The mixture was stirred for 24 h. The solvent was removed under reduced pressure. The addition of 1 mL of MeOH led to the precipitation of the product. The precipitate was centrifuged off and dried under reduced pressure yielding the desired product as a white solid (44 mg, 78.0 µmol) in 78 % yield.

Single crystals for X-ray crystallography were grown from a saturated methylene chloride solution via gas phase diffusion with pentane at room temperature.

^{1}H NMR (600 MHz, CD_2Cl_2, 300 K) δ[ppm] = 7.42 (td, *J* = 7.5, 1.4 Hz, 1H, H-12), 7.36 – 7.24 (m, 7H, H-6,-7,-11,-12,-18,-23,-24), 7.24 – 7.17 (m, 4H, H-10,-13,-22), 6.98 (ddd, *J* = 7.6, 3.3, 1.8 Hz, 1H, H-5), 6.57 (d, *J* = 8.4 Hz, 1H, H-17 or H-19), 6.49 – 6.45 (d, *J* = 8.4 Hz, 1H, H-19 or H-17), 3.49 (s, 3H, -OMe), 3.25 (s, 3H, -OMe), 1.64 (s, 3H, H-1).

^{13}C NMR (151 MHz, CD_2Cl_2, 300 K) δ[ppm] = 168.20 (C-2), 158.12 (C-16 or C-20), 158.06 (C-20 or C-16), 151.39 (d, *J* = 19.1 Hz, C-3), 144.49 (d, *J* = 248.0 Hz, C-26), 141.86 (d, *J* = 35.3 Hz, C-14), 141.28 (d, *J* = 253.2 Hz, C-28), 137.81 (dd, *J* = 671.4, 250.5 Hz, C-27), 136.63 (d, *J* = 11.6 Hz, C-21), 136.51 (C-5), 136.30 (d, *J* = 9.4 Hz, C-9), 134.59 (d, *J* = 1.8 Hz, C-10), 134.45 (d, *J* = 21.7 Hz, C-22), 133.85 (d, *J* = 19.7 Hz, C-4), 132.00 – 131.87 (m, C-7/C-13), 129.68 (C-18), 129.40 (C-12), 129.04 (C-24), 128.70 (d, *J* = 7.3 Hz, C-23), 127.67 (C-11), 126.57 (C-6), 121.08 (C-8), 118.81 (d, *J* = 7.5 Hz, C-15), 112.65 (t, *J* = 19.2 Hz, C-25), 103.98 (C-17 or C-19), 103.61 (C-19 or C-17), 55.60 (-OMe), 55.21 (-OMe), 20.00 (d, *J* = 2.2 Hz, C-1).

^{19}F NMR (376 MHz, CD_2Cl_2, 300 K) δ[ppm] = -139.49 – -139.92 (m, F-29), -154.53 (t, *J* = 20.8 Hz, F-31), -161.94 – -162.37 (m, F-30).

^{31}P NMR (202 MHz, CD_2Cl_2, 300 K) δ[ppm] = -22.53.

Elem. Anal. found (calcd) for (C_{34} H_{24} F_5 O_4 P): C, 65.87 (65.60); H, 4.28 (3.89).

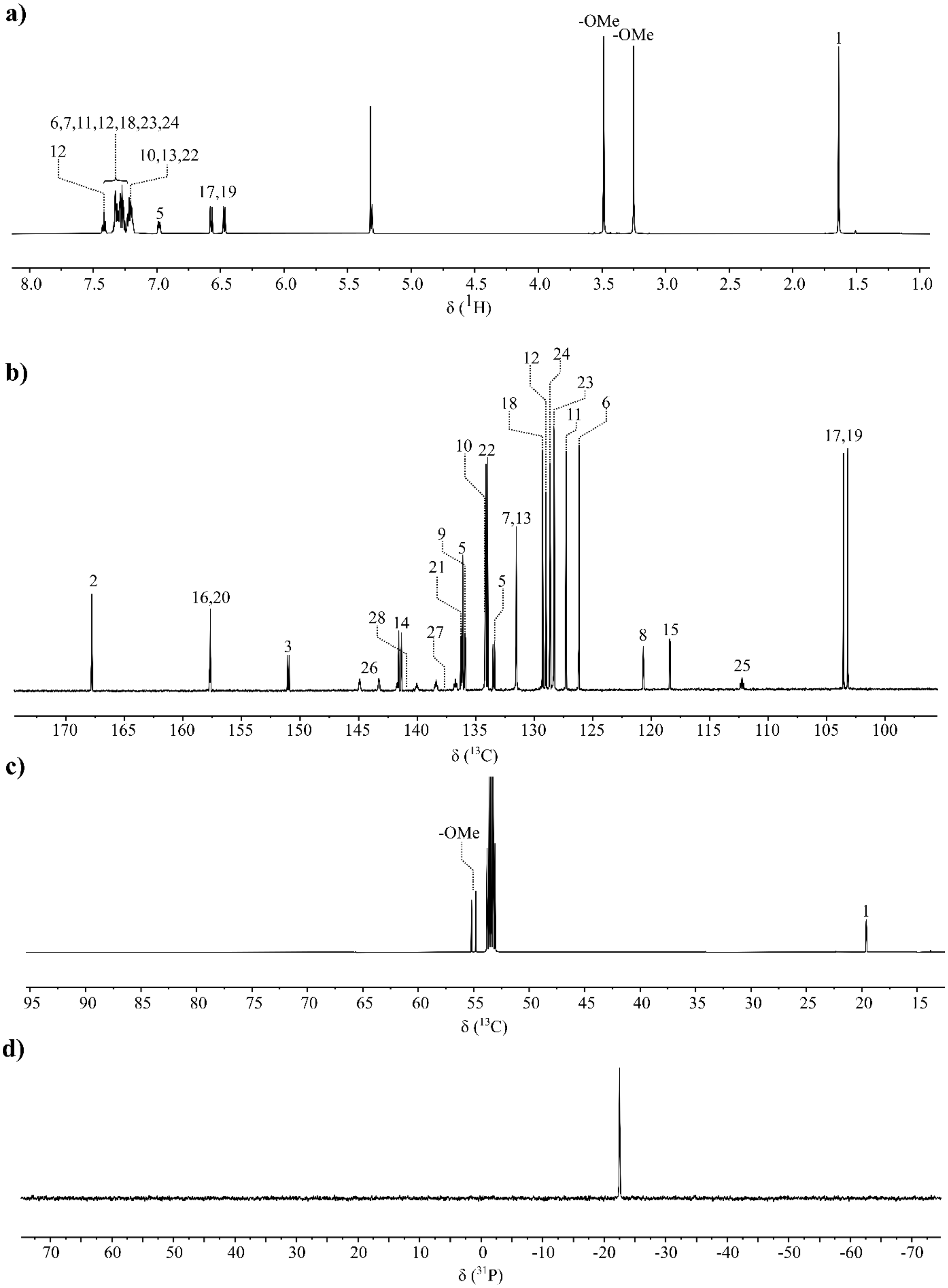

Figure S10. NMR data (25 °C, CD_2Cl_2) of compound **4**. **a:** ^1H NMR spectrum. **b:** ^{13}C NMR spectrum low field region. **c:** ^{13}C NMR spectrum high field region. **d)** ^{31}P NMR spectrum.

Compound **5**:

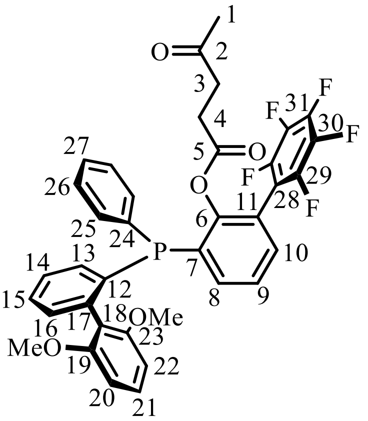

To a solution of **1'** (1 eq, 34.5 µmol, 20 mg) and dimethylaminopyridine (10 mol%, 3.5 µmol, 0.4 mg) in 0.5 mL of dry pyridine levulinic anhydride (2 eq, 68.9 µmol, 14.6 mg) was added at room temperature. The mixture was stirred for 24 h. The solvent was removed under reduced pressure. The addition of 1 mL of MeOH led to the precipitation of the product. The precipitate was centrifuged off and dried under reduced pressure yielding the desired product as a white solid (17 mg, 23.9 µmol) in 69 % yield.

^{1}H NMR (800 MHz, CD_2Cl_2, 300 K) δ[ppm] = 7.43 (td, *J* = 7.5, 1.3 Hz, 1H, H-15), 7.36 – 7.25 (m, 7H, H-9,-10,-14,-15,-21,-26,-27), 7.25 – 7.18 (m, 4H, H-13,-16,-25), 6.97 (ddd, *J* = 7.7, 3.4, 1.7 Hz, 1H, H-8), 6.57 (dd, *J* = 8.4, 0.8 Hz, 1H, H-20 or -22), 6.49 (dd, *J* = 8.4, 0.8 Hz, 1H, H-22 or -20), 3.47 (s, 3H, -OMe), 3.31 (s, 3H, -OMe), 2.28 (t, *J* = 6.5 Hz, 2H, H-4), 2.11 (t, *J* = 6.5, 2H, H-3), 1.95 (s, 3H, H-1).

^{13}C NMR (201 MHz, CD_2Cl_2, 300 K) δ[ppm] = 206.00 (C-2), 170.00 (C-5), 158.13 (C-19 or. C-23), 158.09 (C-23 or C-19), 151.23 (d, *J* = 18.5 Hz, C-6), 144.50 (d, *J* = 247.4 Hz, C-29), 141.94 (d, *J* = 35.4 Hz, C-17), 141.48 (d, *J* = 253.7 Hz, C-31), 138.19 (d, *J* = 248.8 Hz, C-30), 136.75 (d, *J* = 11.8 Hz, C-24), 136.49 – 136.04 (m, C-8/C-12), 134.71 (C-13), 134.42 (d, *J* = 21.5 Hz, C-25), 133.47 (d, *J* = 19.7 Hz, C-7), 131.88 (m, C-10/C-16), 129.65 (C-21), 129.39 (C-15), 129.05 (C-27), 128.72 (d, *J* = 7.3 Hz, C-26), 127.69 (C-14), 126.56 (C-9), 121.41 (C-11), 118.82 (d, *J* = 7.8 Hz, C-18), 112.27 (t, *J* = 21.2 Hz, C-28), 104.02 (C-20 or C-22), 103.63 (C-22 or C-20), 55.58 (-OMe), 55.32 (-OMe), 37.51 (C-4), 29.62 (C-1), 27.40 (C-3).

^{19}F NMR (753 MHz, CD_2Cl_2, 300 K) δ[ppm] = -138.51 – -143.14 (m, F-29), -156.09 (t, *J* = 20.8 Hz, F-31), -159.49 – -168.60 (m, F-30).

^{31}P NMR (202 MHz, CD_2Cl_2, 300 K) δ[ppm] = -22.30.

Elem. Anal. found (calcd) for (C_{37} H_{28} F_5 O_5 P): C, 65.57 (65.49); H, 4.49 (4.16).

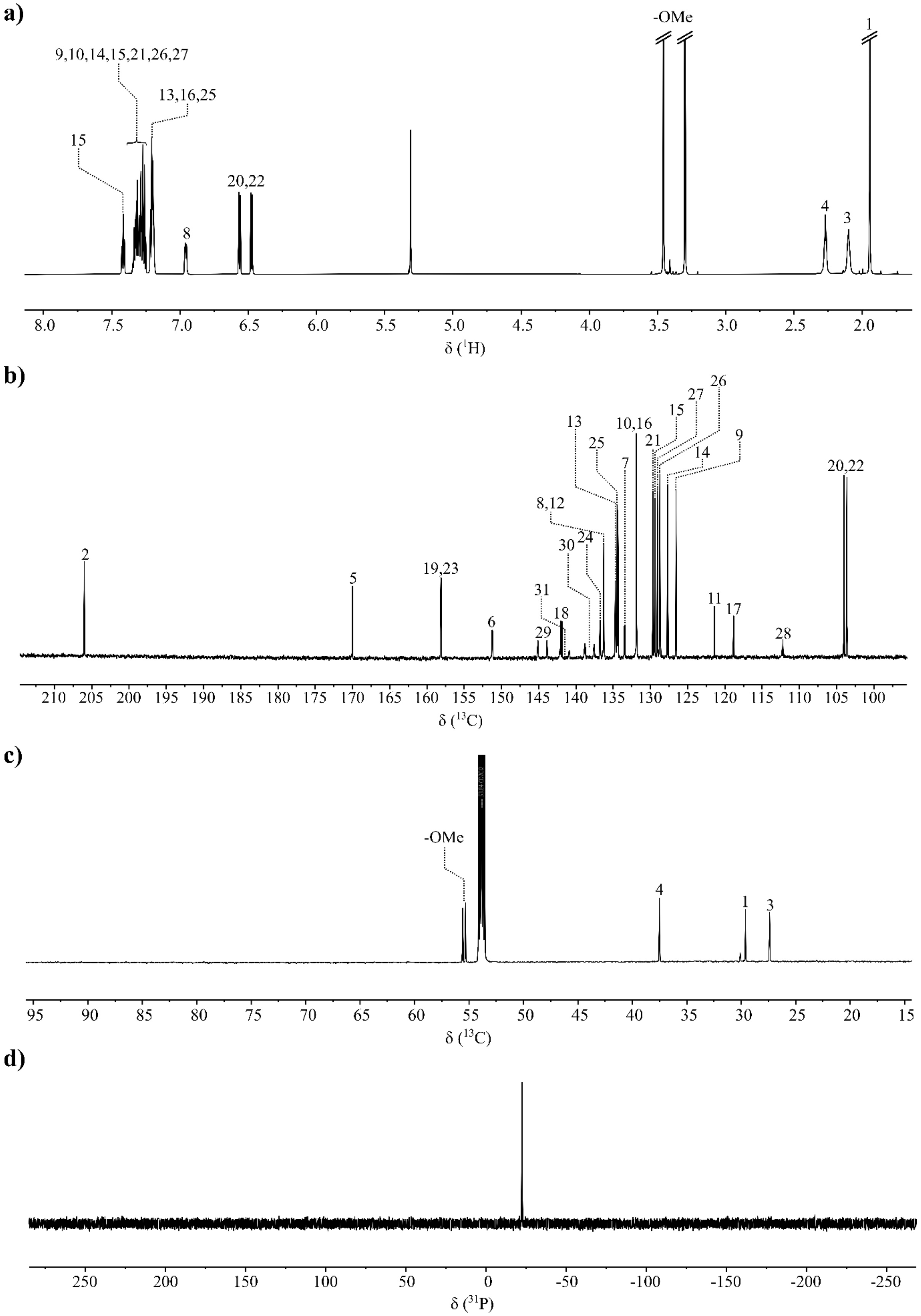

Figure S11. NMR data (25 °C, CD_2Cl_2) of compound **5**. **a:** ^{1}H NMR spectrum. **b:** ^{13}C NMR spectrum low field region. **c:** ^{13}C NMR spectrum high field region. **d)** ^{31}P NMR spectrum.

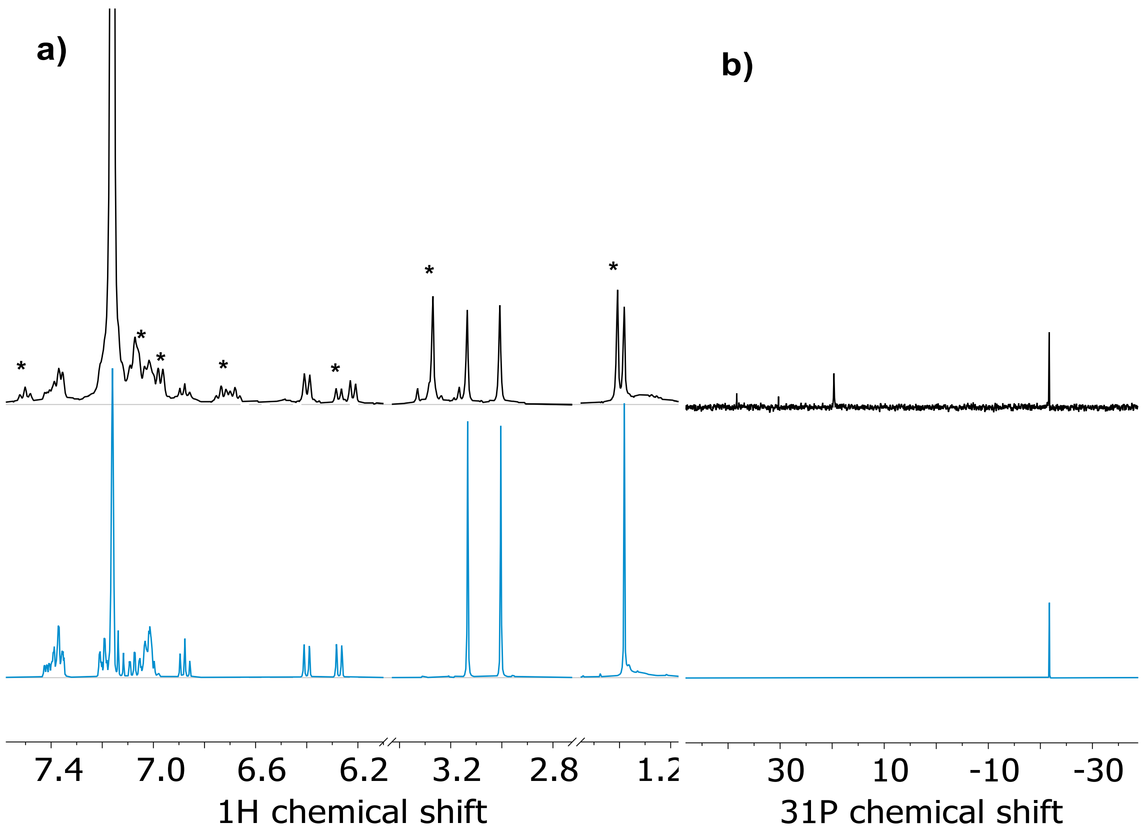

Figure S12. NMR spectra (25 °C, C_6D_6) of the reaction mixture of **1** with CO after 5 d (top, black) and independently synthesized **4** (bottom, blue). **a)** 1H-NMR spectra **b)** ^{31}P-NMR spectra. *Signals arising from unreacted compound **3**.

5. Single crystal analysis

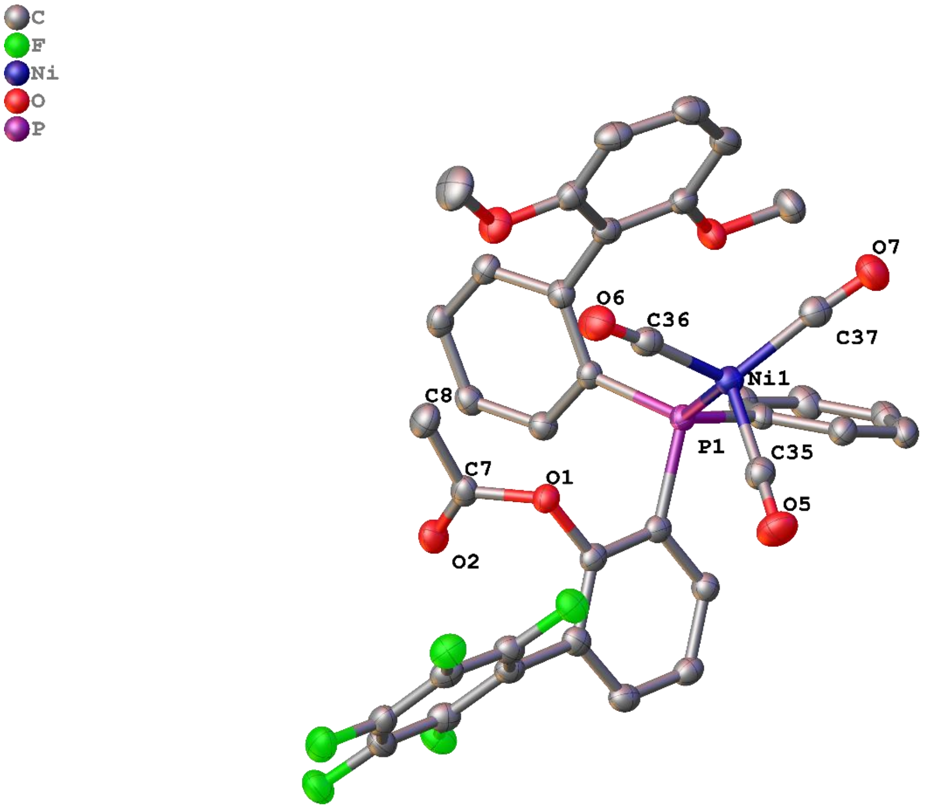

Figure S13. Solid state crystal structure of compound **3**. Thermal ellipsoids drawn at 50% probability level (Hydrogen atoms are omitted for clarity).

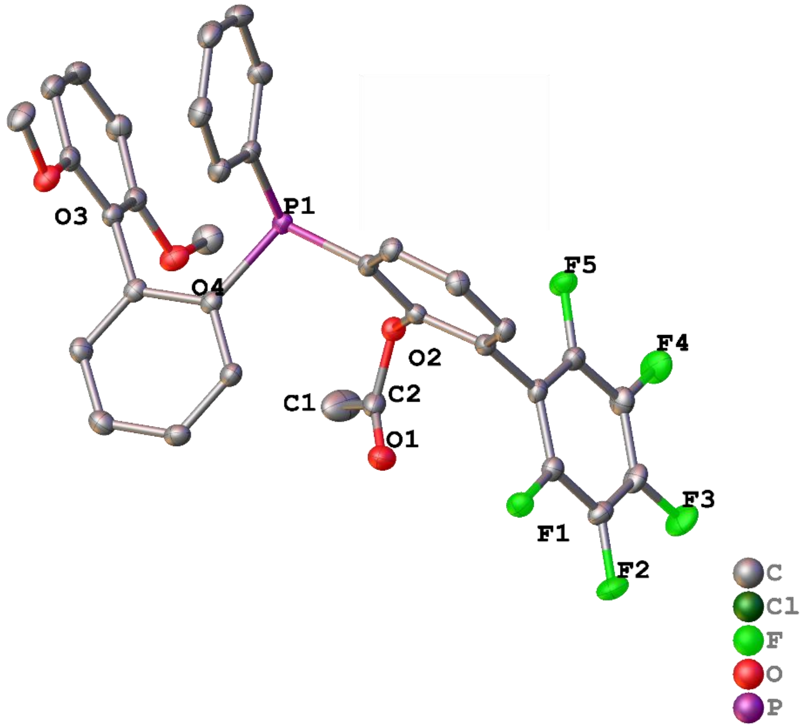

Figure S14. Solid state crystal structure of compound **4** .Thermal ellipsoids drawn at 50% probability level (Hydrogen atoms and cocrystallized methylenechloride are omitted for clarity).

	**Compound 3**	**Compound 4**
Identification code	CCDC2394858	CCDC2394859
Empirical formula	$C_{38}H_{26}Cl_2F_5NiO_7P$	$C_{35}H_{26}Cl_2F_5O_4P$
Formula weight	850.17	707.43
Temperature/K	100	100
Crystal system	triclinic	triclinic
Space group	P-1	P-1
a/Å	10.2854(3)	10.1652(5)
b/Å	11.4664(3)	11.7486(6)
c/Å	16.4516(5)	13.8609(7)
α/°	96.948(2)	107.733(4)
β/°	93.270(2)	92.224(4)
γ/°	110.337(2)	91.096(4)
Volume/Å^3	1795.68(9)	1574.69(14)
Z	2	2
ρ_{calc}g/cm^3	1.572	1.492
μ/mm^{-1}	0.810	0.327
F(000)	864.0	724.0
Crystal size/mm^3	0.35 × 0.317 × 0.3	0.5 × 0.373 × 0.12
Radiation	Mo Kα (λ = 0.71073)	Mo Kα (λ = 0.71073)
2Θ range for data collection/°	3.832 to 53.662	4.948 to 56.06
Index ranges	-12 ≤ h ≤ 13, -14 ≤ k ≤ 14, -20 ≤ l ≤ 20	-13 ≤ h ≤ 13, -12 ≤ k ≤ 15, -18 ≤ l ≤ 18
Reflections collected	21884	17595
Independent reflections	7589 [R_{int} = 0.0184, R_{sigma} = 0.0178]	7448 [R_{int} = 0.0149, R_{sigma} = 0.0152]
Data/restraints/parameters	7589/0/490	7448/0/427
Goodness-of-fit on F^2	1.059	1.052
Final R indexes [I>=2σ (I)]	R_1 = 0.0339, wR_2 = 0.0807	R_1 = 0.0423, wR_2 = 0.1021
Final R indexes [all data]	R_1 = 0.0433, wR_2 = 0.0893	R_1 = 0.0511, wR_2 = 0.1105
Largest diff. peak/hole / e Å^{-3}	0.41/-0.37	0.56/-0.7

6. Pressure reactor experiments

Procedure: Ethylene-carbon monoxide co-polymerizations were conducted in a *Büchi ecoclave* reactor with a 600 mL vessel. The reactor was equipped with a heating and cooling jacket connected to a thermostat, a mechanical stirrer (*Büchi Cyclone c075dc)*, a nitrogen/vacuum supply, a thermocouple dipping into the reaction mixture, and a liquid dosing Pump (Knauer P 4.1s). A *Bronkhorst* MassFlow apparatus consisting of two flow meters (up to 20 g h^{-1} and 200 g h^{-1} ethylene), a pressure meter, and a compressed air-driven badger valve was used to work under constant pressure. All gas valves and devices were connected to a *HiTec Zang LabBox* and operated by *HiTec Zang LabVision®* software (ver. 2.13). Prior to the polymerization experiment, the reactor was evacuated and heated up (thermostat temperature: 90 °C). When the internal reactor temperature was above 60 °C, the reactor was flushed with nitrogen and evacuated three times. Then the temperature was adjusted to the desired reaction temperature. 200 mL of dry and degassed toluene were added, stirring was started at 1000 rpm and the system was equilibrated for 5 min to reach the desired (internal) temperature. The system was pressurized with ^{13}C labeled carbon monoxide (starting from 1 bar nitrogen pressure, adding 0.5 bar of CO). The CO supply was disconnected, and the system was pressurized to an overall pressure of 10 bar with ethylene via a mass flow regulator. The precatalyst (20 µmol) was dissolved in 4 mL of toluene and added to the reactor via the liquid dosing pump with 10 mL per minute (reaction time was started at begin of addition). The tubing of the pump was flushed with toluene for 3 min to assure the complete addition of the precatalyst. A constant pressure was maintained by feeding of ethylene for 60 minutes, and the reactor was depressurized. The solution was added to 400 mL of methanol and stirred for 10 minutes. The formed precipitate was removed via filtration (<30 mg, for ^{13}C NMR characterization see Figure S11) and the solvent of the filtrate was removed under reduced pressure. The residue was dried under vacuum (~0.01 mbar) and characterized by NMR spectroscopy.

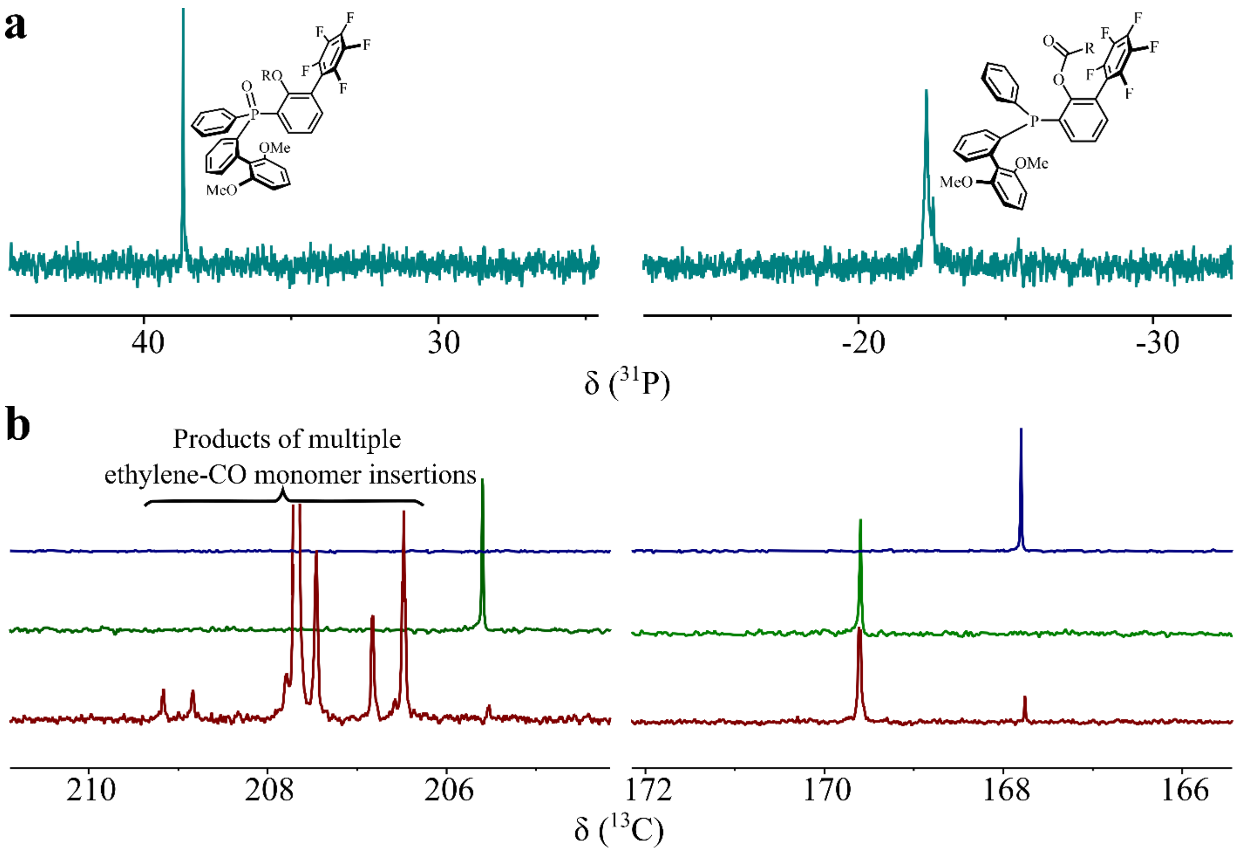

Figure S15. NMR spectroscopic analysis of decomposition products from pressure reactor experiments. **a:** ^{31}P NMR spectrum (25 °C, CD_2Cl_2) of decomposition products from preparative reactor experiments identifying the acylated ligand species as well as oxidized phosphine ligands. **b:** ^{13}C NMR spectrum (25 °C, CD_2Cl_2) of species **4** (top), **5** (center) as a reference, and the decomposition products of preparative reactor experiments.

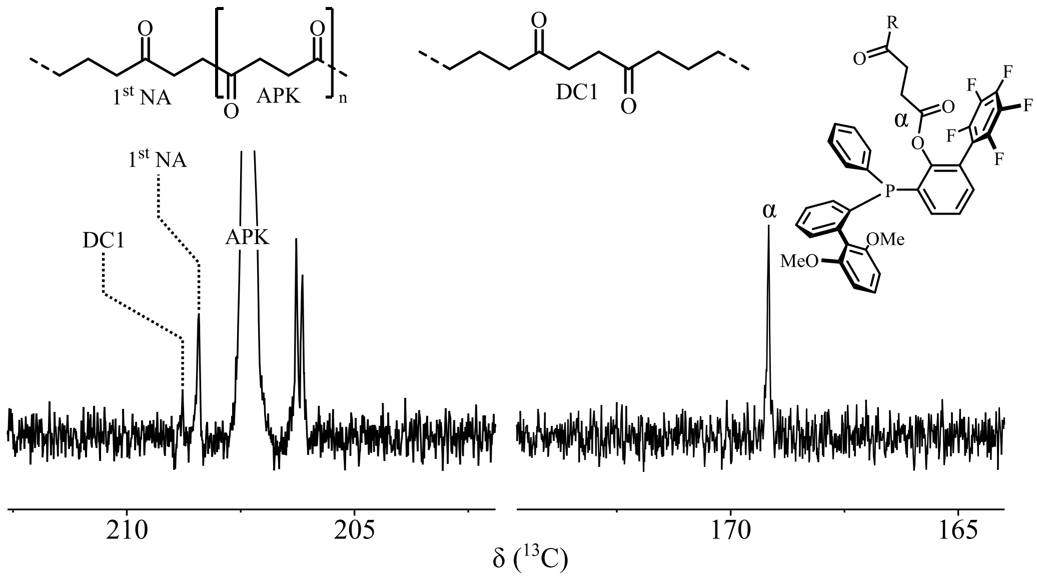

Figure S16. Details of ^{13}C NMR spectroscopy (110 °C, $C_2D_2Cl_4$) of the precipitate from a pressure reactor experiment.

(1) Dolomanov, O. V.; Bourhis, L. J.; Gildea, R. J.; Howard, J. A. K.; Puschmann, H. OLEX2: a complete structure solution, refinement and analysis program. J. Appl. Crystallogr. 2009, 42, 339–341.
(2) Sheldrick, G. M. SHELXT - Integrated space-group and crystal-structure determination. Acta Cryst. A 2015, 71, 3–8.
(3) Sheldrick, G. M. Crystal structure refinement with SHELXL. Acta Cryst. C, 2015, 71, 3–8.
(4) Bourhis, L. J.; Dolomanov, O. V.; Gildea, R. J.; Howard, J. A. K.; Puschmann, H. The anatomy of a comprehensive constrained, restrained refinement program for the modern computing environment - Olex2 dissected. Acta Cryst. A 2015, 71, 59–75.
(5) Zhang, Y.; Mu, H.; Wang, X.; Pan, L.; Li, Y. Elaborate Tuning in Ligand Makes a Big Difference in Catalytic Performance: Bulky Nickel Catalysts for (Co)polymerization of Ethylene with Promising Vinyl Polar Monomers. ChemCatChem, 11(9), 2329-2340 2019.
(6) Macchione, G.; Maza, S.; Mar Kayser, M.; Paz, J. L. de; Nieto, P. M. Synthesis of Chondroitin Sulfate Oligosaccharides Using N -(Tetrachlorophthaloyl)- and N -(Trifluoroacetyl)galactosamine Building Blocks. Eur. J. Org. Chem. 2014, 2014 (18), 3868–3884.
(7) Goodwin, R. D. Toluene thermophysical properties from 178 to 800 K at pressures to 1000 bar. *Journal of Physical and Chemical Reference Data* **1989**, *18* (4), 1565-1636.
(8) Cargill, R. W. *Solubility Data Series: Carbon Monoxide*; Elsevier, 2013.
